# Fluconazole-Resistant and Virulence-Associated Yeasts from the Vulva: Evidence of a Potential Reservoir

**DOI:** 10.3390/jof12020106

**Published:** 2026-02-03

**Authors:** Maria Margarida Silva, Mariana Zagalo Fernandes, Sofia Moura, Ana Sofia Esteves, Ana Sofia Oliveira, Carlos Gaspar, José Martinez-de-Oliveira, Ana Palmeira-de-Oliveira, Joana Rolo

**Affiliations:** 1 RISE-Health, Department of Medical Sciences, Faculty of Health Sciences, University of Beira Interior (UBI), Av. Infante D. Henrique, 6200-506 Covilhã, Portugalmarianamzf@hotmail.com (M.Z.F.);; 2 Faculty of Health Sciences, University of Beira Interior (UBI), Av. Infante D. Henrique, 6201-001 Covilhã, Portugal; 3 Department of Clinical Pathology, Santarém District Hospital, Lezíria Local Healthcare Unit, 2005-177 Santarém, Portugal; 4 Labfit-HPRD—Health Products Research and Development Lda, 6200-284 Covilhã, Portugal

**Keywords:** *Candida*, epidemiology, genital tract infections, virulence factors, women

## Abstract

Vulvovaginal candidosis is an affliction caused by yeasts. Symptoms in the vulva are generally associated with the spreading of infected vaginal fluid. To better understand the role of the vulva in these dynamics, in this study we aim to fully identify and characterize non-*Candida albicans* vulvar yeast isolates. Fifty-four vulvar swabs were obtained from 31 women attending a gynecological consultation. After species identification, fluconazole susceptibility was assessed by the microdilution broth method. Biofilm biomass was quantified using crystal violet staining, and phospholipase and hemolysin production were assessed by plating a calibrated suspension in suitable culture media. Finally, adherence to cervical cells was assessed by infecting a monolayer of HeLa cells. Among the 54 vulvar isolates obtained, 12 different species were identified. About 54% (29/54) of vulvar isolates were resistant to fluconazole. All isolates were able to produce a high amount of biofilm biomass. *Pichia kudriavzevii* and *Rhodotorula mucilaginosa* were the only species that produced phospholipase; hemolysin production was detected in isolates belonging to almost all species. Almost all species had the ability to adhere to HeLa cells. These results indicate that the vulva act as a reservoir for fluconazole-resistant yeasts, which are also potentially virulent.

## 1. Introduction

In recent years, there has been an increase in the incidence of human invasive fungal infections, possibly related to the overall increase in populations at risk [1,2]. Furthermore, climate change has altered the geographic regions where fungal species were endemic, leading to an increase in emergent fungal diseases [3]. Nonetheless, studies focusing on fungal diseases are scarce compared with the available data for bacterial and viral diseases [2]; as a consequence, the true incidence, mortality, and morbidity caused by fungal infections are difficult to assess. In an effort to stimulate research on fungal diseases, the World Health Organization (WHO) published the first list of fungal priority pathogens [1], focusing on species that are more frequently isolated in human infections and that are frequently resistant to antifungals. In the critical group, the highest ranking member of the WHO fungal priority pathogens list is *Candida albicans*, which is overall the most prevalent yeast isolated in human samples [1]. The treatment of fungal diseases is often challenging, since few treatment options are available, and few novel treatments are being generated as treatment options [4]. Azole compounds are among the most used antifungals, but resistance rates appear to be increasing among some species [1].

Although associated with a myriad of different human infections, many fungal species are also commensal to humans, mostly on the skin, gut, oral, and vaginal mucosae [2]. The female genital tract, namely the vagina and the vulva, is colonized by different yeast species, of which the most frequent is *Candida albicans* [5]. *C. albicans* and other *Candida* species have been implicated in vulvovaginal candidosis, an infection of the vulva and/or the vagina that occurs frequently worldwide, particularly in women of child-bearing age [6]. Previously, it was thought that infection of the vulva occurred concomitantly with vaginal infection, caused by the resulting increase and spread of vaginal fluid [6]. However, a recent study by our research group has revealed that infection of the vulva may occur independently of vaginal infection and that non-*Candida* species like *Rhodotorula mucilaginosa* are involved, being identified in 51.5% of the 33 samples in our study [7]. Species of the genus *Rhodotorula* can be recovered from the human skin, nails, and respiratory and gastrointestinal tracts [8]. These species also have invasive potential, being described as the etiological agent in 0.5% to 2.3% of all cases of fungemia described in the USA and Europe, which assumes an estimated mortality of 12–20% [9]. Patients with *Rhodotorula* spp. must be treated with a systemic antifungal therapy; however, these yeasts appear to be intrinsically resistant to most azoles [7] and echinocandins, so further studies are needed to find adequate therapies. Other yeast species that have been emerging in infections of the female genital tract are *Trichosporon* spp. [10] and non-*neoformans Cryptococcus* [11], on which much less is known. In this study, we aim to contribute to the characterization of the mycobiota of the female genital tract by studying vulvar non-*C. albicans* yeasts.

## 2. Materials and Methods

### 2.1. Isolation of Yeast Clinical Isolates

Thirty women were retrospectively involved in this study, instead of being specifically recruited. Vulvar samples were collected for diagnostic purposes between 2019 and 2023 by an expert gynecologist in Vila do Conde, Portugal. Immediately after collection, the swabs were streaked in a Sabouraud Dextrose agar plate (SDA, VWR, Radnor, PA, USA) and incubated at room temperature (22–25 °C). After recording the diagnostic results, the culture plates were anonymized and transported to the research laboratory. After subculturing in SDA, the samples were stored at −80 °C in Brain Heart Infusion broth (BHI, VWR, Radnor, PA, USA) supplemented with 20% glycerol (Fisher Scientific, Hudson, NH, USA). Demographic and clinical data were provided by the practitioner. This protocol has been approved by an ethics committee (University of Beira Interior, Portugal, CE-UBI-Pj-2018-022). After arrival at the research lab, isolates were subcultured on CHROMAgar Candida ID (Biomérieux, Marcy-l’Étoile, France). Only cultures that presented non-green colonies indicative of non-*Candida albicans* were included in this study. A total of 54 non-*C. albicans* vulvar isolates were obtained from these samples and further characterized. The cultures that yielded green colonies on CHROMAgar Candida ID culture media were further characterized by PCR amplification of the hyphal wall protein 1 (HWP1) gene, using primers that allow discrimination between *Candida africana*, *Candida albicans*, and *Candida dubliniensis*. Three different DNA fragments are produced using these primers: approximately 700 bp for *C. africana*, 941 bp for *C. albicans*, and 569 bp for *C. dubliniensis* [12]. The colonies that yielded green-blue colonies, that failed to be amplified, or that resulted in non-specific bands were identified by Vitek 2 or MALDI-tof as *C. tropicalis*. These isolates were not included in the study.

### 2.2. Subculturing and Purification of Different Yeast Species

The optimal growth temperature was determined for each isolate by subculturing twice in SDA, and incubating in duplicate at 37 °C and 25 °C for 48 h. The temperature at which a consistent lawn culture was observed was selected for further assays (Table S1).

### 2.3. Species Identification

Species identification was performed by a semi-automated method, which took into account 46 biochemical reactions (Vitek 2, Biomerieux Genomic Marcy-l’Étoile, France), and confirmed by DNA sequencing. DNA from each yeast isolate was extracted using the NZY Tissue gDNA Isolation kit (NZYTech, Lda., Lisboa, Portugal). An internal fragment of the D1/D2 domain of the 28S rRNA gene was amplified by PCR using the NL1 (GCATATCAATAAGCGGAGGAAAAG) and NL4 (GGTCCGTGTTTCAAGACGG) primers, as described by Leaw et al. (2006) [13]. Amplification products were purified using Speedy NZY ExoSAP Mix (NZYTech, Lda., Lisboa, Portugal), and sequencing was performed at STABVida, Lisboa, Portugal. Resulting sequences were compared against the NCBI database using BLAST for species identification, considering a >95% nucleotide sequence identity threshold [14] (https://blast.ncbi.nlm.nih.gov/Blast.cgi, accessed on 19 May 2025) and ≥90% coverage. The nucleotide sequences were deposited in GenBank (https://www.ncbi.nlm.nih.gov/genbank/, accessed on 8 January 2026): accession numbers PX842637-PX842683.

### 2.4. Determination of Antifungal Susceptibility

Susceptibility to the most prescribed antifungal in the gynecological practice—fluconazole [15]—was tested by the microdilution broth assay, following the protocol previously described by Fernandes et al. (2023) [7], and EUCAST guidelines. Briefly, fluconazole (Sigma-Aldrich, St. Louis, MO, USA) susceptibility was assessed in 96-well plates with concentrations ranging from 2 to 64 μg/mL. Plates were incubated for 18–24 h at 37 °C or at room temperature. For comparison purposes, the resistance threshold was standardized to 4 μg/mL.

### 2.5. Assessment of the Ability to Form Biofilms

Biofilm formation was assessed following the protocol described by Sherry et al. (2017) [16] and adapted by Faria-Gonçalves et al. (2020) [17]. Briefly, the isolates were cultured in SDA, adjusted to 0.5 McFarland in RPMI-1640 and inoculated (100 µL) in 96-well microplates, incubating for 24 h at 37 °C or at room temperature. After removal of planktonic cells, the biofilms were washed with PBS 1× (PBS: 1.37 M NaCl, Fisher Scientific, Hudson, NH, USA; 27 mM KCl, ChemLab, Zedelgem, Belgium; 100 mM Na_2_HPO_4_, Fisher Scientific, Hudson, NH, USA; 20 mM KH_2_PO_4_, ChemLab, Zedelgem, Belgium), fixed with methanol (Fisher Scientific, Hudson, NH, USA), and stained with crystal violet (VWR, Avantor, Radnor, PA, USA). The bound dye was solubilized with 33% acetic acid and quantified by absorbance reading at 590 nm. A 1:10 dilution was prepared in 33% acetic acid before absorbance reading to ensure readings between 0.1 and 0.9. Two independent assays were performed in quadruplicate for each isolate.

### 2.6. Assessment of Phospholipase Production

Phospholipase activity was assessed by using egg yolk Sabouraud Dextrose Agar (SDA) medium, as previously described [17]. Plates were incubated at 37 °C (or room temperature) for five days and visually examined after incubation; they were considered positive when a precipitation halo surrounding the colony was observed. Halo diameters were measured using a ruler and reported in millimeters (mm). Enzymatic activity (Pz) was determined by calculating the ratio between the colony’s diameter and the diameter of the colony plus the precipitation zone. Thus, the smaller the Pz value, the higher the enzymatic activity: Pz = 1.0 (no enzymatic activity), 0.64 < Pz > 1.0 (moderately positive enzymatic activity), and Pz ≤ 0.63 (strongly positive enzymatic activity).

### 2.7. Assessment of Hemolysin Production

To assess the hemolytic activity of the isolates, two independent assays were performed. Blood agar plates composed of Columbia agar (5% sheep blood, Frilabo, Porto, Portugal), a medium commonly used to determine hemolytic reactions, were used. First, the isolates were prepared by streaking onto SDA medium (VWR, Radnor, PA, USA) and incubating at 37 °C (or room temperature) for 24 h. After 24 h, two to three colonies from each prepared inoculum were collected and resuspended in 2 mL of 0.85% NaCl, adjusted to an optical density (OD) of 0.5 McFarland. A 10 μL drop of this suspension was placed onto each blood agar plate, which was then incubated at 37 °C (or room temperature) for 48 h (Binder, Neckarsulm, Germany). After incubation, the plates were visually examined and considered positive when a clear halo surrounding the inoculum was observed.

### 2.8. Assessment of the Ability to Adhere to HeLa Cells

A representative isolate of each species was randomly selected to assess adherence to HeLa cells (ATCC^®^ CCL-2^TM^) [18]. The cells were maintained as monolayers in T-flasks using Dulbecco’s Modified Eagle Medium (DMEM, Sigma-Aldrich, St. Louis, MO, USA), supplemented with 10% heat-inactivated fetal bovine serum (FBS; Gibco, Thermo Fisher Scientific, Hudson, NY, USA), 1.5 g/L sodium bicarbonate (Gibco, Thermo Fisher Scientific, Hudson, NY, USA), and a mixture of penicillin (100 U/mL) and streptomycin (100 μg/mL) (Sigma-Aldrich, St. Louis, MO, USA). Cultures were incubated at 37 °C in a humidified atmosphere of 5% CO_2_ for 24–48 h until full differentiation. The culture medium was refreshed every 2–3 days. Once confluence was reached, cells were subcultured weekly following the supplier’s recommendations. HeLa cells (2 × 10^5^ cells per well) were then seeded into 6-well plates and incubated for 24 h at 37 °C to achieve confluence. Yeast cells were suspended in DMEM supplemented with 2% FBS and adjusted to 0.5 McFarland (≈1 × 10^6^ cells/mL per well). The monolayers were infected for 2 h at 37 °C to allow adhesion. Following incubation, the wells were washed twice with 1 mL of sterile PBS 1× to remove non-adherent yeasts. Adherent yeasts were detached by adding 1 mL of 0.01% Trypsin-EDTA (VWR, Avantor, Radnor, PA, USA). From the resulting suspension, 10 μL were transferred to a sterile 96-well plate containing 90 μL of sterile PBS, in quadruplicate. Serial tenfold dilutions (1:10 to 10^−4^) were performed, and 5 μL of each dilution was plated onto SDA plates in duplicate. The plates were incubated at 37 °C (or room temperature) for 24 h, after which colony-forming units (CFU) were enumerated.

### 2.9. Statistical Analysis

Statistical analysis was performed to assess the association between the clinical state of patients and species distribution by using GraphPad Prism version 8.0 (Informer Technologies, Inc., Los Angeles, CA, USA)and applying the variance test (chi-square test). Statistical significance was defined as *p* < 0.05 for two-sided tests. In addition, to assess if differences between species were significant, one-way ANOVA was used (non-parametric test, Kruskal–Wallis test). Furthermore, the statistical significance was set at *p*-values < 0.05.

## 3. Results

### 3.1. Yeast Species Distribution

**Figure 1:**
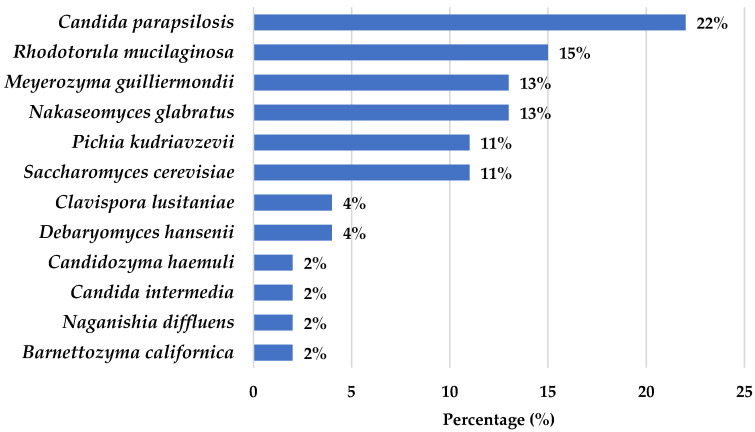
Relative frequency of non-*C. albicans* yeast species identified in vulvar samples. Percentages represent the proportion of each species within the total isolated yeasts.

A total of 54 non-*C. albicans* vulvar isolates were included in the study, representing 12 distinct yeast species (Figure 1). *Candida parapsilosis* was the most frequent species (22%), followed by *Rhodotorula mucilaginosa* (15%), *Nakaseomyces glabratus* (13%), and *Meyerozyma guilliermondii* (13%). In contrast, *Candidozyma haemuli*, *Candida intermedia*, *Naganishia diffluens*, and *Barnettozyma californica* were recovered only once among our samples (2% each).

The non-*C. albicans* vulvar isolates were obtained from 30 women. For nine of these women, more than one isolate was recovered (Table 1). When this species distribution was compared with the clinical state of the women (Table 1), we found that *Saccharomyces cerevisiae* was found exclusively among vulvar samples obtained from women with a clinical diagnosis of vulvovaginal candidosis, being the species more frequently retrieved from this clinical context (27%, *p* < 0.05), while *M. guilliermondii* was found exclusively in women who were asymptomatic at the sampling time, being also a species more frequent in this context (30%, *p* < 0.05). *C. parapsilosis* and *Pichia kudriavzevii* were also more associated with a symptomatic state—taking into account samples recovered both from VVC cases and cases of women with other vulvovaginal afflictions (*p* < 0.05).

### 3.2. Susceptibility to Azole Compounds

Fluconazole susceptibility was evaluated for all vulvar yeast isolates based on the fluconazole concentration required to inhibit at least 50% of fungal growth. The distribution of values across species is presented in Table 2. Overall, 29 out of 54 isolates (54%) required concentrations above 4 µg/mL. Moreover, all isolates of *R. mucilaginosa* (*n* = 8), *P. kudriavzevii* (*n* = 6), *D. hansenii* (*n* = 2), *N. diffluens* (*n* = 1), and *B.a californica* (*n* = 1) exhibited MIC_50_ values above 4 µg/mL. In contrast, all isolates of *S. cerevisiae* (*n* = 6), *C. intermedia* (*n* = 1), *C. haemuli* (*n* = 1), and *C. lusitaniae* (*n* = 2) were consistently inhibited at lower concentrations.

Strain variability for some species was also observed. For instance, among *N. glabratus*, four of seven isolates (57%) required above 4 µg/mL, whereas three (43%) remained susceptible. A similar pattern was observed in *M. guilliermondii*, with six of seven isolates (86%) showing reduced susceptibility and one (14%) inhibited at lower concentrations. Regarding *C. parapsilosis*, although a small percentage of the recovered isolates 8% (1/12) showed resistance to the antifungal, the vast majority demonstrated fluconazole susceptibility 92% (11/12).

### 3.3. Phenotypic Expression of Virulence Factors

Phospholipase activity was generally low among the vulvar isolates (Table 3). Only two species demonstrated measurable enzymatic production, namely *P. kudriavzevii* and *R. mucilaginosa*, in which 66.67% (4/6) and 13% (1/8) of isolates of the respective species exhibited moderate activity. All remaining species displayed exclusively absent phospholipase activity (Pz = 1.0).

Hemolysin production was more widespread, with all isolates of *C. intermedia*, *C. parapsilosis*, *C. haemuli*, *C. lusitaniae*, *N. glabratus*, *M. guilliermondii*, and *R. mucilaginosa* being hemolysin-positive. The isolates of the remaining species showed no hemolysin activity (*B. californica*, *D. hansenii*, *N. diffluens* and *S. cerevisiae*).

**Figure 2:**
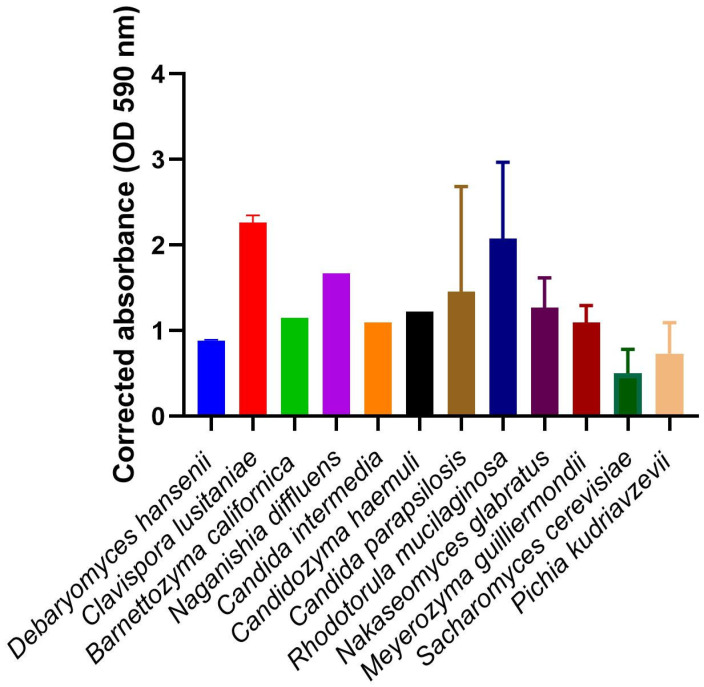
Corrected absorbance values (OD 590 nm) of crystal violet-stained biofilm biomass. The average obtained for all isolates of each vulvar species is shown, as well as the standard deviation. The differences observed between species were found to be statistically significant (*p* < 0.05).

The biofilm-forming ability of all isolates was quantified through crystal violet staining, and the mean absorbance values for each species, along with the corresponding standard deviation, are presented in Figure 2. Statistical analysis revealed that the differences observed between species were significant (*p* < 0.05). Based on the absorbance measurements, *C. lusitaniae* (Abs = 2.263) and *R. mucilaginosa* (Abs = 2.077) were the species that produced the largest amount of biofilm biomass compared to the other species, followed by *N. diffluens* (Abs = 1.668) and *C. parapsilosis* (Abs = 1.458). In contrast, *S. cerevisiae* was the species that produced the lowest amount of biomass (Abs = 0.502). Marked intra-species variability was observed for some taxa. Notably, the mean obtained among isolates belonging to *C. parapsilosis* and *R. mucilaginosa* showed high standard deviations (1.227 and 0.892, respectively), indicating substantial heterogeneity in biofilm production among isolates of these species.

**Figure 3:**
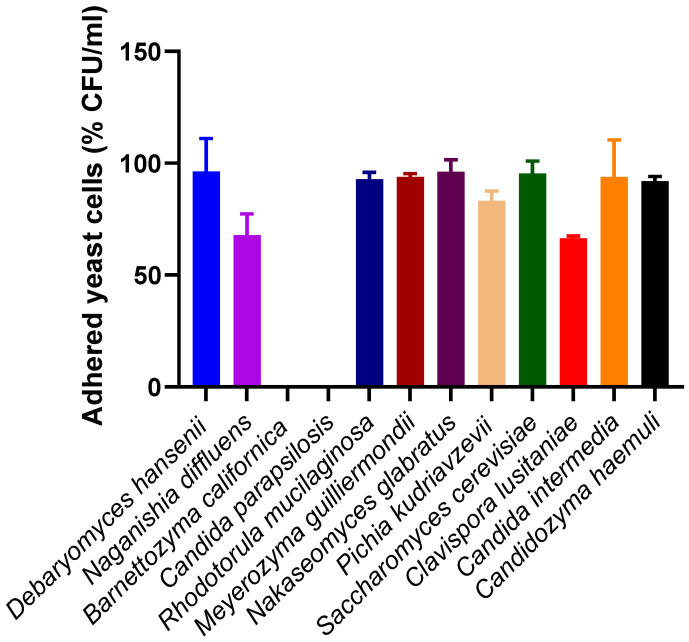
Adhered yeast cells (% of initial suspension, CFU/mL). The results are shown for one isolate of each species. The differences observed between species were found to be statistically non-significant (*p* > 0.05).

Adhesion to HeLa cells by one isolate of each vulvar species was characterized by quantifying the number of recovered microorganisms (CFU/mL) after 2 h of infection. The percentage of the initial suspension that adhered to HeLa cells is presented in Figure 3. Almost all species exhibited high adhesion capacity, above 90%. Lower adhesion was observed for *P. kudriavzevii* (83.2%), *N. diffluens* (67.8%), and *C. lusitaniae* (66.5%), although these differences were found to be statistically non-significant (*p* > 0.05). A representative image of adhered yeasts is shown in Supplementary Figure S1. Noticeably, *C. parapsilosis* and *B. californica* isolates showed no ability to adhere (0%).

## 4. Discussion

In recent decades, the number of fungal infections, particularly those caused by yeasts, has increased worldwide [1,4]. *Candida albicans* remains the most frequently isolated yeast from human samples and is currently considered a top-priority fungal pathogen by the WHO [1]. Nevertheless, other yeast species are increasingly recognized as important human pathogens, despite being comparatively understudied [2]. Vulvovaginal candidiasis is the second most prevalent vaginal infection among women of reproductive age, affecting millions of women globally [19,20]. Species of the genus *Candida* and other yeasts can cause infections both in the vulva (vulvitis) and in the vagina (vaginitis), which may occur independently or simultaneously; however, in both cases, they are generally referred to as vulvovaginal candidiasis [5,6]. Classically, vulvar infection was considered to originate as a consequence of vaginal infection due to the spread of infected vaginal fluid. However, results from recent studies have revealed a group of symptomatic patients from whom yeasts were isolated exclusively from the vulva, without signs of vaginal infection [7]. Nevertheless, although these are considered two complex niches, there is currently little information available regarding the spatial distribution and characteristics of yeasts isolated in these two niches [7]. Given this context, fungal infections constitute a significant public health challenge, making faster diagnosis and more appropriate and effective treatment necessary to reduce associated complications.

In this study, the prevalence of different non-*C. albicans* species was determined, and the phenotypic peculiarities of yeasts originating from the vulva were characterized, assessing the pathophysiological potential of isolates from the vulvar mycobiota. This evaluation focused on determining the susceptibility profiles of these isolates to fluconazole, their ability to form biofilm, their production of extracellular enzymes, and also their ability to adhere to a cervical cell line. In particular, a high diversity of yeast species was observed. The most prevalent species was *Candida parapsilosis* (22%), followed by *Rhodotorula mucilaginosa* (15%), *Nakaseomyces glabratus* (13%), and *Meyerozyma guilliermondii* (13%). Overall, the most prevalent yeast species recovered were common vaginitis pathogens, which have been extensively reported [6,7,21,22]: *C. parapsilosis*, *N. glabratus* (former *C. glabrata*), *P. kudriavzevii* (former *C. krusei*), *M. guilliermondii* (former *C. guilliermondii*), *S. cerevisiae*, *C. lusitanea*, *D. hansenii* (former *C. famata*), and *R. mucilaginosa*. Regarding the species for which we recovered a single isolate (*B. californica*, *N. diffluens*, *C. intermedia*, and *C. haemuli*), *N. diffluens*, *C. intermedia*, and *C. haemuli* have been recently isolated from cases of skin infections, demonstrating their virulence potential [23,24,25]. To our knowledge, our study is the first to describe the isolation of *B. californica* from a human sample. In previous studies, the most prevalent non-*C. albicans* species in vulvar samples were *Rhodotorula* spp. followed by *C. parapsilosis* [7], showing partial agreement with our findings.

Fluconazole is one of the most frequently administered antifungals for the treatment of yeast infections caused by species of the genus *Candida* [15,26]. Fluconazole susceptibility testing revealed high levels of resistance among our non-*Candida albicans* vulvar isolates (54%). Several species showed consistent non-susceptibility, particularly *Rhodotorula mucilaginosa*, *Pichia kudriavzevii*, *Debaryomyces hansenii*, *Barnettozyma californica*, and *Naganishia*. *R. mucilaginosa* showed MIC values higher than 64 μg/mL, *P. kudriavzevii* showed MIC values ranging from 16 μg/mL to 32 μg/mL, *D. hansenii* from 4 μg/mL to 8 μg/mL, and *B. californica* and *N. diffluens* exhibited MIC values of 4 μg/mL. Despite these four species exhibiting antifungal resistance, it is evident that *R. mucilaginosa* displays the highest minimum inhibitory concentration values. Fernandes et al. (2023) evaluated the activity of fluconazole against 30 isolates of *Rhodotorula* spp., of which 27 showed MIC values equal to or above 64 μL/mL, while the remaining isolates displayed values between 16 μL/mL and 64 μL/mL [7].

Seifi et al. (2013) identified and isolated 69 *Rhodotorula* spp. isolates from two hospitals and assessed their susceptibility to fluconazole; all isolates were resistant, including six *R. mucilaginosa* isolates [27]. Similarly, Mokhtar et al. (2024) reported an MIC of 128 μL/mL for a *R. mucilaginosa* isolate [28]. Overall, in vitro studies have consistently demonstrated that *Rhodotorula* spp. exhibits intrinsic resistance to fluconazole and echinocandins. However, greater susceptibility has been observed to other azoles, such as voriconazole and itraconazole [28]. In contrast, all isolates of *Saccharomyces cerevisiae*, *Candida intermedia*, *Candidozyma haemuli*, and *Clavispora lusitaniae* had minimum inhibitory concentration values ≤2 μg/mL. For *N. glabratus* and *M. guilliermondii*, a larger proportion of isolates showed an increased resistance to fluconazole, with MIC values of 4 μg/mL and 32 μg/mL, and 4 μg/mL to 8 μg/mL, respectively. In *C. parapsilosis*, although a small percentage of isolates displayed resistance to the antifungal (MIC ≥ 64 μg/mL), the majority exhibited antifungal susceptibility. Existing studies evaluating antifungal susceptibility in non-*Candida albicans* species have demonstrated reduced susceptibility to azole compounds [29]. In Portugal, it was observed that all isolates of *C. tropicalis* and *C. parapsilosis* obtained from the vulva exhibited minimum inhibitory concentration values equal to or lower than 2 μg/mL [7,22]. However, due to the lack of specific data on vulvar isolates, when comparing the results obtained with studies that used vulvovaginal samples, the literature reports that a proportion of *N. glabratus* isolates are known to exhibit resistance to fluconazole, with a slight tendency to increase over recent years [7,22]. For other European countries, it was not possible to find studies conducted specifically on isolates from the vulva, which hinders comparisons of the results obtained and limits contextualization with other European regions.

Over the past decades, fungal infections associated with biofilm formation have shown an incidence of 65% of all recorded cases [30]. Biofilm growth is one of the growth modes of several opportunistic fungi [31], providing various advantages to yeasts, such as increased resistance to antimicrobial agents and antifungal treatments [32], strengthening virulence factors and enhancing their ability to invade host tissues [5,32]. In the present study, the biofilm-forming capacity of the isolates was assessed by quantifying total biofilm biomass with crystal violet staining. *C. lusitaniae* produced the highest amount of biofilm, followed by *R. mucilaginosa*, *N. diffluens*, and *C. parapsilosis.* On the other hand, the species that produces the lowest amount of biofilm biomass was *S. cerevisiae.* However, the literature indicates that biofilm formation is highly dense for *N. glabratus* and downplays biofilm formation in *C. parapsilosis* [16,17,33], which does not align with the results obtained in this study. Possibly, the increase in biofilm formation ability could be a specific adaptation of the yeasts to the vulvar ecological niche.

The production of hydrolytic enzymes, such as phospholipases, and hemolytic enzymes facilitates the supply of nutrients and promotes fungal access to host tissues [5]. While the expression of phospholipases uses phospholipids present in human and animal cell membranes as substrates [5], hemolytic activity reflects the yeast’s ability to destroy erythrocytes in order to obtain iron through the production of substances known as hemolysins [34], thus conferring an important virulence factor for the establishment of infection [5]. Among our isolates, phospholipase activity was low, detected in only 9.3% (5/54) of the isolates. The only species exhibiting enzymatic activity were *P. kudriavzevii*, and *R. mucilaginosa*, with isolates showing moderate enzymatic activity. Previous reports have detailed that non-*C. albicans* species such as *C. glabrata*, *C. tropicalis*, *C. sake*, and *C. parapsilosis* can produce phospholipases [35,36,37]. Several reports have described the ability of *R. mucilaginosa* to express phospholipases [38]. It has been reported for *R. mucilaginosa* isolates that the ones derived from humans are more likely to exhibit phospholipase activity than those from environmental or animal sources [39]. The low proportion of phospholipase producers found in our study may raise the hypothesis that vulvar samples phenotypically do not express phospholipases, which could be linked with their poor invasive potential. On the other hand, we found a high proportion of hemolytic isolates. In a study conducted by Rörig et al. (2009), among non-*Candida albicans* species, only *C. parapsilosis* exhibited hemolysin production [40]. However, the highly frequent enzymatic activity observed in this study is quite significant, therefore conferring an adaptive advantage for the establishment of infections in the host. According to Pendrak et al. (2004), this activity may be involved in mechanisms of phenotypic changes related to the induction of a more virulent phenotype or resistance to host defenses [41].

Finally, regarding adhesion to HeLa cells, we found that most species had a high ability to adhere in vitro to these cells, which demonstrates the capacity of these yeast species to adapt to the host. In contrast, *C. parapsilosis* and *B. californica* did not show any adhesion capacity. Based on the available literature, no studies were found that address or challenge these results, highlighting the need for further investigations related to fungal infections.

As expected for genital samples, we were able to recover consecutive isolates from the same women. Recurrent vaginitis caused by yeast is common, affecting 10% of all women experiencing VVC, and isolation of more than one species at a time is also common [21,42]. Of note, in our study, *S. cerevisiae* was found exclusively among vulvar samples obtained from women with a clinical diagnosis of vulvovaginal candidosis. However, in our study, the isolates were also susceptible to fluconazole, did not produce extracellular enzymes, and had a poor biofilm formation ability. Nevertheless, they had a high ability to adhere to cervical cells. This species is occasionally reported in cases of vaginitis [22,43], and despite its non-virulent profile, surveillance is advised. *C. parapsilosis* and *P. kudriavzevii* were also more associated with a symptomatic state. In our study, vulvar isolates from these species revealed a more virulent profile, being resistant to fluconazole and able to produce extracellular enzymes, biofilms, and adherence to cervical cells. These species are recognized vaginal pathogens, being reported as common agents of vaginitis [44,45,46].

The present study has some limitations. The results obtained from vulvar sample isolates are restricted to patients attending a private gynecology clinic, which means they are not representative of the national population. Furthermore, there is limited scientific research on the distribution of non-*C. albicans* species in the vulvar niche, as well as on the virulence factors associated with them, making comparisons with the results obtained challenging.

## 5. Conclusions

In recent years, studies in the field of human mycobiota have shown significant expansion. Although studies related to the vaginal mycobiota have increased, the same ratio has not occurred for the vulvar mycobiota. Despite the close anatomical proximity of these two niches, they represent quite distinct environments for microorganisms, highlighting the need for more studies on the distribution of species present in the vulvar environment and their impact on women’s vaginal health. However, understanding vulvar microbial ecology may be complicated by the anatomy of the vulva, which is likely not a single niche but rather a structure composed of multiple microbial habitats. Therefore, this study aimed to determine the prevalence of different isolated species and to characterize the metabolic peculiarities of yeasts originating from the vulva, assessing the pathophysiological potential of isolates belonging to the vulvar mycobiota. In addition, by focusing on non-*Candida albicans* isolates, we aim to provide further knowledge on these cryptic yeast species. We found a high species diversity, where fluconazole-resistant isolates were frequent. Furthermore, we found a high frequency of hemolytic isolates, with a high ability to form biofilms and adhere to cervical cells. Taken together, our findings highlight the complexity and phenotypic variability of the isolated yeasts. This underscores the importance of continuous monitoring of antifungal resistance and a deeper understanding of virulence factors in order to support the development of more effective therapeutic strategies.

In the future, it is crucial to understand the patterns of these infections at both national and international levels by conducting additional studies to further analyze the role of virulence factor expression, as well as the distribution of non-*C. albicans* species in the vulvar niche. This highlights the importance and need to more deeply explore the impact of these species on the establishment of colonies and fungal infection in the lower female genital tract.

## Figures and Tables

**Table 1:** Species distribution of vulvar yeast isolates considering the clinical status of the patients. The data show the number of isolates in each clinical category, followed by their relative frequency within that column (%). VVC: vulvovaginal candidosis. Other vulvovaginal affliction: other symptomatic state related with a vulvovaginal affliction. Values highlighted in bold reveal differences that were found to be statistically significant (*p* < 0.05).

Species	Asymptomatic	VVC	Other Vulvovaginal Affliction	Total
*Candida parapsilosis*	3 (14%)	5 (23%)	4 (44%)	12
*Rhodotorula mucilaginosa*	4 (17%)	1 (5%)	3 (34%)	8
*Nakaseomyces glabratus*	2 (9%)	4 (18%)	1 (11%)	7
*Meyerozyma guilliermondii*	**7 (30%)**	**0 (0%)**	**0 (0%)**	**7**
*Saccharomyces cerevisiae*	**0 (0%)**	**6 (27%)**	**0 (0%)**	**6**
*Pichia kudriavzevii*	2 (9%)	4 (18%)	0 (0%)	6
*Debaryomyces hansenii*	0 (0%)	2 (9%)	0 (0%)	2
*Clavispora lusitaniae*	2 (9%)	0 (0%)	0 (0%)	2
*Barnettozyma californica*	1 (4%)	0 (0%)	0 (0%)	1
*Naganishia diffluens*	1 (4%)	0 (0%)	0 (0%)	1
*Candida intermedia*	1 (4%)	0 (0%)	0 (0%)	1
*Candidozyma haemuli*	0 (0%)	0 (0%)	1 (11%)	1
Total	23 (100%) ^a^	22 (100%) ^b^	9 (100%) ^c^	54

^a^ Obtained from 12 women (for three women, more than one isolate was recovered); ^b^ obtained from 11 women (for four women, more than one isolate was recovered); ^c^ obtained from 7 women (for two women, more than one isolate was recovered).

**Table 2:** Distribution of the isolates among each species, according to the minimum inhibitory concentration to fluconazole (MIC_50_, µg/mL).

Species	*n*	Fluconazole MIC (µg/mL)					
		≤2	4	8	16	32	≥64
*Candida parapsilosis*	12	91.67%	0%	0%	0%	0%	8.33%
*Rhodotorula mucilaginosa*	8	0%	0%	0%	0%	0%	100%
*Nakaseomyces glabratus*	7	42.86%	14.29%	0%	0%	42.86%	0%
*Meyerozyma guilliermondii*	7	14.29%	71.43%	14.29%	0%	0%	0%
*Saccharomyces cerevisiae*	6	100%	0%	0%	0%	0%	0%
*Pichia kudriavzevii*	6	0%	0%	0%	33.33%	66.67%	0%
*Debaryomyces hansenii*	2	0%	50%	50%	0%	0%	0%
*Clavispora lusitaniae*	2	100%	0%	0%	0%	0%	0%
*Barnettozyma californica*	1	0%	100%	0%	0%	0%	0%
*Naganishia diffluens*	1	0%	100%	0%	0%	0%	0%
*Candida intermedia*	1	100%	0%	0%	0%	0%	0%
*Candidozyma haemuli*	1	100%	0%	0%	0%	0%	0%

**Table 3:** Frequency of enzymatic activity of vulvar isolates belonging to each species. Phospholipase activity is shown according to the Pz value obtained (absent, moderate, strong). Hemolysin activity is shown as positive or negative.

Species	*n*	Phospholipase Activity (Pz)			Hemolysin Activity	
		Absent	Moderate	Strong	Positive	Negative
*Candida parapsilosis*	12	100%	0%	0%	100%	0%
*Rhodotorula mucilaginosa*	8	87.5%	13%	0%	100%	0%
*Meyerozyma guilliermondii*	7	100%	0%	0%	100%	0%
*Nakaseomyces glabratus*	7	100%	0%	0%	100%	0%
*Pichia kudriavzevii*	6	33.33%	66.67%	0%	0%	100%
*Saccharomyces cerevisiae*	6	100%	0%	0%	0%	100%
*Clavispora lusitaniae*	2	100%	0%	0%	100%	0%
*Debaryomyces hansenii*	2	100%	0%	0%	0%	100%
*Barnettozyma californica*	1	100%	0%	0%	0%	100%
*Candida intermedia*	1	100%	0%	0%	100%	0%
*Candidozyma haemuli*	1	100%	0%	0%	100%	0%
*Naganishia diffluens*	1	100%	0%	0%	0%	100%

## Data Availability

All data is presented in the manuscript. The nucleotide sequences were deposited in GenBank (https://www.ncbi.nlm.nih.gov/genbank/, accessed on 8 January 2026): accession numbers PX842637-PX842683.

## Supplementary Materials

The following supporting information can be downloaded at: https://www.mdpi.com/article/10.3390/jof12020106/s1, Figure S1. HeLa cells after infection with yeast species. A. Control (no infection). B. *Pichia kudriavzevii*. C. *Rhodotorula mucilaginosa*. Photos obtained with an optical microscope (400×, Olympus XI, Olympus Corporation, Hachioji-shi, Tokyo, Japan). Table S1. Optimal growth temperature (°C) of each isolate examined in the study.
