# Supplementary material for: Fluconazole-Resistant and Virulence-Associated Yeasts from the Vulva: Evidence of a Potential Reservoir
Source: J Fungi (Basel). 2026 Feb 3;12(2):106. doi: 10.3390/jof12020106 (PMC12941488; doi:10.3390/jof12020106)
Supplement: Supplementary file 1 [file jof-12-00106-s001.zip › jof-4045863-supplementary.pdf]

Supplementary Material

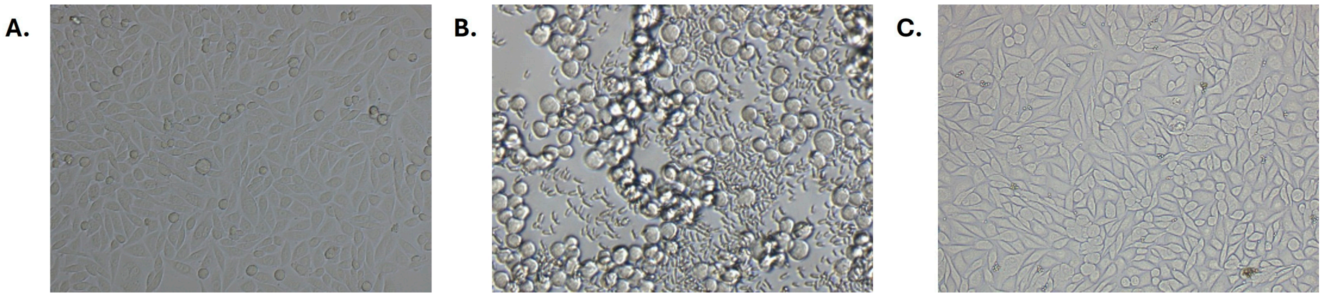


Figure S1. HeLa cells after infection with yeast species. A. Control (no infection). B. *Pichia kudriavzevii*. C. *Rhodotorula mucilaginosa*. Photos obtained with an optical microscope (400x, Olympus XI)

Table S1. Optimal growth temperature (ºC) of each isolate examined in the study.

| Isolate | Species | Optimal growth temperature (ºC) |
|---|---|---|
| **V1** | *Saccharomyces cerevisiae* | 37 |
| **V2** | *Nakaseomyces glabratus* | 37 |
| **V3** | *Saccharomyces cerevisiae* | 37 |
| **V4** | *Saccharomyces cerevisiae* | 37 |
| **V5** | *Saccharomyces cerevisiae* | 37 |
| **V6** | *Saccharomyces cerevisiae* | 37 |
| **V7** | *Saccharomyces cerevisiae* | 37 |
| **V10** | *Barnettozyma californica* | 25 |
| **V12** | *Pichia kudriavzevii* | 37 |
| **V13** | *Candida parapsilosis* | 37 |
| **V14** | *Pichia kudriavzevii* | 37 |
| **V15** | *Naganishia diffluens* | 25 |
| **V16** | *Candida parapsilosis* | 37 |
| **V17** | *Pichia kudriavzevii* | 37 |
| **V18** | *Candida intermedia* | 25 |
| **V19** | *Meyerozyma guilliermondii* | 37 |
| **V20** | *Candida parapsilosis* | 37 |
| **V21** | *Debaryomyces hansenii* | 25 |
| **V22** | *Debaryomyces hansenii* | 25 |
| **V23** | *Meyerozyma guilliermondii* | 37 |
| **V24** | *Meyerozyma guilliermondii* | 37 |
| **V25** | *Meyerozyma guilliermondii* | 37 |
| **V27** | *Meyerozyma guilliermondii* | 37 |
| **V28** | *Meyerozyma guilliermondii* | 37 |
| **V29** | *Candidozyma haemuli* | 25 |
| **V30** | *Candida parapsilosis* | 37 |
| **V31** | *Meyerozyma guilliermondii* | 37 |
| **V32** | *Candida parapsilosis* | 37 |
| **V33** | *Clavispora lusitaniae* | 25 |
| **V34** | *Candida parapsilosis* | 37 |
| **V35** | *Candida parapsilosis* | 37 |
| **V36** | *Pichia kudriavzevii* | 37 |
| **V37** | *Pichia kudriavzevii* | 37 |

| | | |
|---|---|---|
| **V38** | *Clavispora lusitaniae* | 25 |
| **V39** | *Nakaseomyces glabratus* | 37 |
| **V40** | *Candida parapsilosis* | 37 |
| **V42** | *Nakaseomyces glabratus* | 37 |
| **V43** | *Candida parapsilosis* | 37 |
| **V45** | *Candida parapsilosis* | 37 |
| **V46** | *Nakaseomyces glabratus* | 37 |
| **V47** | *Candida parapsilosis* | 37 |
| **V48** | *Pichia kudriavzevii* | 37 |
| **V49** | *Nakaseomyces glabratus* | 37 |
| **V50** | *Candida parapsilosis* | 37 |
| **V51** | *Nakaseomyces glabratus* | 37 |
| **V52** | *Nakaseomyces glabratus* | 37 |
| **R1** | *Rhodotorula mucilaginosa* | 25 |
| **R2** | *Rhodotorula mucilaginosa* | 25 |
| **R3** | *Rhodotorula mucilaginosa* | 25 |
| **R4** | *Rhodotorula mucilaginosa* | 25 |
| **R5** | *Rhodotorula mucilaginosa* | 25 |
| **R7** | *Rhodotorula mucilaginosa* | 25 |
| **R8** | *Rhodotorula mucilaginosa* | 25 |
| **R10** | *Rhodotorula mucilaginosa* | 25 |
